# A99 HIGHER HOSPITAL INPATIENT RACIAL DIVERSITY IS ASSOCIATED WITH BETTER OUTCOMES AMONG HISPANIC AND INDIGENOUS AMERICAN PATIENTS FOR FIVE COMMON GASTROINTESTINAL DIAGNOSES

**DOI:** 10.1093/jcag/gwad061.099

**Published:** 2024-02-14

**Authors:** B Lawendy, M Sedarous, O Babajide, A Adekunle, M Rubens, P Okafor

**Affiliations:** Western University, London, ON, Canada; Queen's University, Kingston, ON, Canada; One Brooklyn Health, Brooklyn, NY; St. Luke’s Hospital, Chesterfield, MO; Miami Cancer Institute, Miami, FL; Mayo Clinic in Florida, Jacksonville, FL

## Abstract

**Background:**

There is evidence that gastrointestinal disease (GI) outcomes are poorer among patients from underrepresented backgrounds. However, the impact of hospital patient racial diversity on GI outcomes is understudied.

**Aims:**

We aimed to investigate the impact of hospital patient racial diversity on GI outcomes

**Methods:**

Using the 2019 National Inpatient Sample (NIS), racial diversity was defined by the percentage of Hispanic or Indigenous American patients discharged from each hospital. GI discharge diagnoses were defined by diagnostic related group. We included GI bleeding, inflammatory bowel diseases, GI obstruction, cirrhosis, and alcoholic hepatitis. Logistic regression was used to predict major complication rates or comorbidity (MCC), long length of stay (LOS), and high total charges. Control variables included age, gender, payer type, patient location, area-associated income quartile, hospital characteristics including size, urban vs. rural, teaching vs. nonteaching, region, and the interaction of the percentage of Hispanic and Indigenous Americans with patient race. Results were validated with the 2018 NIS dataset.

**Results:**

Our analysis cohort of 537,830 hospitalizations included 252,225 GI bleeding discharges, 59,310 gastrointestinal obstruction discharges, 106,820 cirrhosis and alcoholic hepatitis discharges, and 119,475 inflammatory bowel diseases discharges. In the unadjusted analyses, MCC rates were higher among Hispanic (24.8%) and Indigenous American patients (30.4%), compared to Whites (18.3%). In adjusted analyses, compared to white patients, Hispanic patients had higher MCC rates [adjusted odds ratio (OR) 1.21, 95% Confidence Interval (CI) 1.15-1.28]. The same trend was seen among Indigenous American patients [OR 1.25, (95% CI) 1.09-1.43]. However, as hospital Hispanic diversity increased, inpatient MCC outcomes for Hispanics improved [OR 0.93, (95% CI) 0.87-1.14], and were even better among Indigenous American patients as hospital inpatient Indigenous American diversity increased [OR 0.83, (95% CI) 0.73-0.94] (Table 1). A similar improvement in MCC with increasing Hispanic and Indigenous American diversity was observed in the 2018 validation cohort. Table 1 highlights impact of increasing diversity on LOS and total charges.

**Conclusions:**

Increasing hospital inpatient Hispanic and Indigenous American diversity is associated with better outcomes for these underrepresented minority groups. More research is needed on the impact of cultural competence and linguistic concordance on patient outcomes.

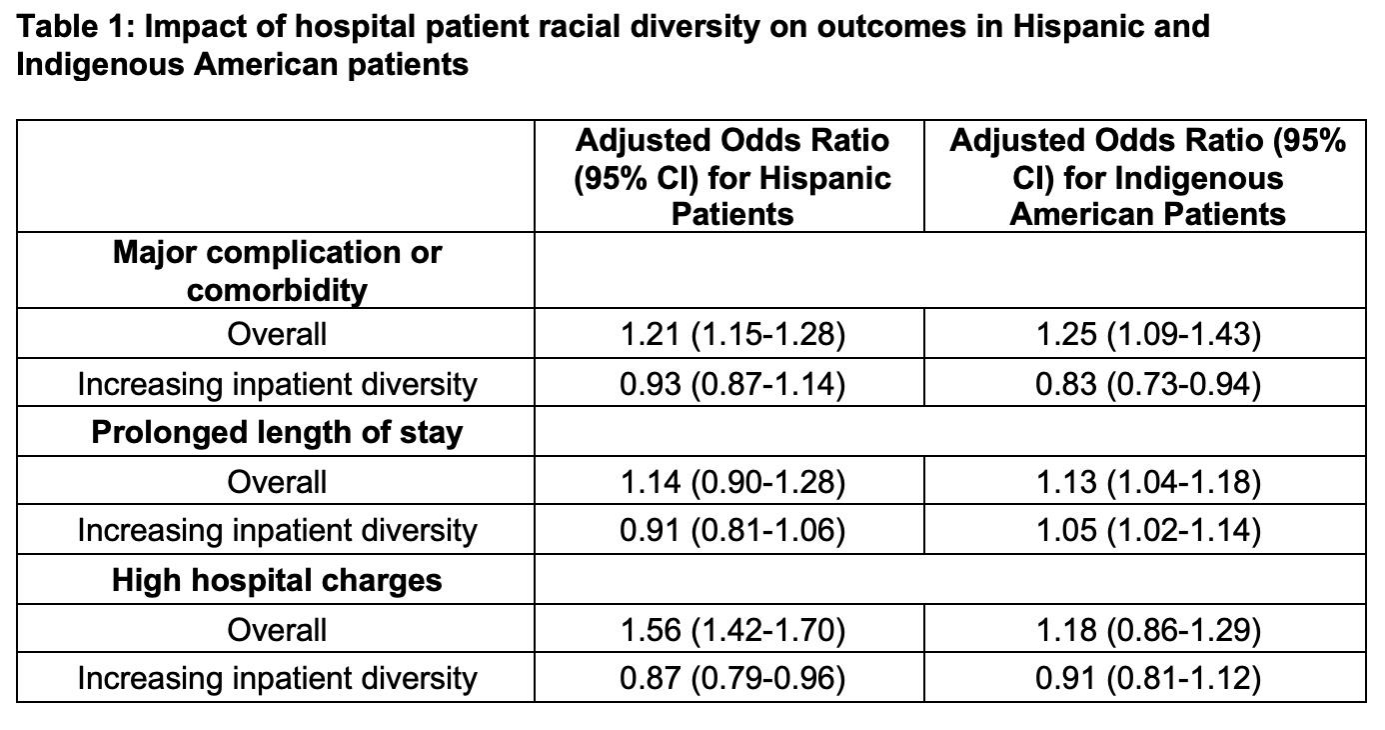

**Funding Agencies:**

None

